# Supplementary material for: In-silico characterization of deleterious non-synonymous SNPs in the human S1PR1 gene reveals structural instability and altered ligand affinity
Source: PLoS One. 2026 Feb 2;21(2):e0339370. doi: 10.1371/journal.pone.0339370 (PMC12863678; doi:10.1371/journal.pone.0339370)
Supplement: S1 Table — (DOCX) [file pone.0339370.s001.docx]

**S1 Table.** Distribution of S1PR1 nsSNP (missense), synonymous, intron, in-frame deletions, in-frame insertions and others.

| **SNP** | **Amount** | **Percentage (%)** |
| --- | --- | --- |
| nsSNP (missense) | 212 | 6.51 |
| Synonymous | 168 | 5.16 |
| Intron | 2215 | 67.96 |
| In-frame deletion | 2 | 0.06 |
| In-frame insertions | 1 | 0.03 |
| Others | 661 | 20.28 |
| Total | 3259 | 100.00 |
