# Supplementary material for: In-silico characterization of deleterious non-synonymous SNPs in the human S1PR1 gene reveals structural instability and altered ligand affinity
Source: PLoS One. 2026 Feb 2;21(2):e0339370. doi: 10.1371/journal.pone.0339370 (PMC12863678; doi:10.1371/journal.pone.0339370)
Supplement: S2 Table — (DOCX) [file pone.0339370.s002.docx]

**S2 Table.** Unanimous deleterious or damaging nsSNPs in the S1PR1 protein predicted by eight different tools.

| **Rs no** | **Amino acid substitution** | **SIFT** | **PolyPhen-2** | **PROVEAN** | **PhD-SNP** | **SNPs & GO** | **META-SNP** | **Predict SNP** | **SNAP** | **PANTHER** |
| --- | --- | --- | --- | --- | --- | --- | --- | --- | --- | --- |
| rs74454232 | T193P | APF | PD | DL | D | D | D | DL | DL | PSD |
| rs149198314 | R120P | APF | PD | DL | D | D | D | DL | DL | PSD |
| rs370425078 | Y198C | APF | PD | DL | D | D | D | DL | DL | PSD |
| rs758342367 | Y81C | APF | PD | DL | D | D | D | DL | DL | PD |
| rs770192267 | I224N | APF | PD | DL | D | D | D | DL | DL | PSD |
|  | I224T | APF | PD | DL | D | D | D | DL | DL | PSD |
|  | I224S | APF | PD | DL | D | D | D | DL | DL | PSD |
| rs780607576 | N307D | APF | PD | DL | D | D | D | DL | DL | PSD |
| rs1021007044 | L61P | APF | PD | DL | D | D | D | DL | DL | PD |
| rs1053857245 | L275P | APF | PD | DL | D | D | D | DL | DL | PSD |
| rs1176228992 | S192T | APF | PD | DL | D | D | D | DL | DL | PSD |
|  | S192P | APF | PD | DL | D | D | D | DL | DL | PSD |
| rs1265245364 | M318T | APF | PD | DL | D | D | D | DL | DL | PSD |
| rs1295820624 | A127D | APF | PD | DL | D | D | D | DL | DL | PSD |
| rs1346744443 | F125S | APF | PD | DL | D | D | D | DL | DL | PSD |
| rs1360246180 | C184Y | APF | PD | DL | D | D | D | DL | DL | PSD |
| rs1399911077 | S131F | APF | PD | DL | D | D | D | DL | DL | PSD |
| rs1412721199 | C328P | APF | PD | DL | D | D | D | DL | DL | PSD |
| rs1459080234 | G122R | APF | PD | DL | D | D | D | DL | DL | PSD |
| rs1557707683 | I173N | APF | PD | DL | D | D | D | DL | DL | PSD |
| rs1570828745 | D91A | APF | PD | DL | D | D | D | DL | DL | PSD |
| rs1570829459 | V258G | APF | PD | DL | D | D | D | DL | DL | PSD |
| rs1652818744 | L212P | APF | PD | DL | D | D | D | DL | DL | PSD |
| rs1652822674 | L254Q | APF | PD | DL | D | D | D | DL | DL | PSD |
| rs1652827524 | A300V | APF | PD | DL | D | D | D | DL | DL | PSD |

Note: APF: Affect Protein Function, PD: Probably Damaging, DL: Deleterious, D: disease, PSD: Possibly Damaging
