# Supplementary material for: In-silico characterization of deleterious non-synonymous SNPs in the human S1PR1 gene reveals structural instability and altered ligand affinity
Source: PLoS One. 2026 Feb 2;21(2):e0339370. doi: 10.1371/journal.pone.0339370 (PMC12863678; doi:10.1371/journal.pone.0339370)
Supplement: S3 Table — (DOCX) [file pone.0339370.s003.docx]

**S3 Table.** Structural impact prediction of S1PR1 high-risk pathogenic (deletorious) nsSNPs of S1PR1 protein.

| **Amino Acid**  **Substitution** | | | **T193P** | **R120P** | **Y198C** | | **Y81C** | **I224N** | **I224T** | **I224S** | **N307D** | **L61P** |
| --- | --- | --- | --- | --- | --- | --- | --- | --- | --- | --- | --- | --- |
| Mupro stability  prediction | | | Decrease | Decrease | Decrease | | Decrease | Decrease | Decrease | Decrease | Decrease | Decrease |
| Imutant2 | | | Increase | Decrease | Decrease | | Decrease | Decrease | Decrease | Decrease | Increase | Decrease |
| NetSurfP 3.0 prediction | Native | RSA | Buried | Buried | Buried | | Buried | Buried | Buried | Buried | Buried | Exposed |
|  |  | RSA (%) | 20 | 7 | 20 | | 8 | 1 | 1 | 1 | 2 | 58 |
|  |  | ASA | 35 | 18 | 53 | | 21 | 3 | 3 | 3 | 4 | 116 |
|  |  | Pdisorder | 0 | 0 | 0 | | 0 | 0 | 0 | 0 | 0 | 0 |
|  | Mutant | RSA | Exposed | Buried | Buried | | Buried | Buried | Buried | Buried | Buried | Exposed |
|  |  | RSA (%) | 29 | 7 | 12 | | 8 | 3 | 3 | 3 | 3 | 56 |
|  |  | ASA | 46 | 11 | 21 | | 13 | 6 | 5 | 5 | 5 | 89 |
|  |  | Pdisorder | 0 | 0 | 0 | | 0 | 0 | 0 | 0 | 0 | 0 |
| mCSM | | | Destabilizing | Destabilizing | Destabilizing | | Destabilizing | Destabilizing | Destabilizing | Destabilizing | Destabilizing | Destabilizing |
| SDM | | | Destabilizing | Destabilizing | Destabilizing | | Destabilizing | Destabilizing | Destabilizing | Destabilizing | Destabilizing | Destabilizing |
| DUET | | | Destabilizing | Destabilizing | Destabilizing | | Destabilizing | Destabilizing | Destabilizing | Destabilizing | Destabilizing | Destabilizing |
| Change in size | | | Decrease | Decrease | Decrease | | Decrease | Increase | Decrease | Decrease | Increase | Decrease |
| Change of charge | | | Unchanged | Positive>Neutral | Unchanged | | Unchanged | Unchanged | Unchanged | Unchanged | Neutral>Negative | Unchanged |
| Change in hydrophobicity | | | Increase | Increase | Increase | | Increase | Decrease | Decrease | Decrease | Unchanged | Unchanged |
| Other impacts | | | The mutation will cause loss of hydrogen bonds in the core of the protein and as a result local conformation will be slightly destabilized. | Wild-type residue forms a salt bridge, disturb the ionic interaction made by the original, wild-type residue. The mutation will cause disrupts an α-helix and severe effects on the structure of the protein. Which causes empty space in the core of the protein. Lost H bond disturb correct folding the protein. | The mutation will cause an empty space in the core of the protein. Lost H bond disturb correct folding | | The mutation will cause an empty space in the core of the protein. Lost H bond disturb correct folding | None | The mutation will cause an empty space in the core of the protein. | The mutation will cause an empty space in the core of the protein. | None | None |
| **Amino Acid**  **Substitution** | | | **L275P** | | **S192T** | | **S192P** | **M318T** | **A127D** | **F125S** | **C184Y** | **S131F** |
| Mupro stability  prediction | | | Decrease | | Decrease | | Decrease | Decrease | Increase | Decrease | Decrease | Increase |
| Imutant2 | | | Decrease | | Decrease | | Increase | Decrease | Decrease | Decrease | Decrease | Increase |
| NetSurfP 3.0 prediction | Native | RSA | Buried | | Buried | | Buried | Buried | Buried | Buried | Buried | Buried |
|  |  | RSA (%) | 8 | | 10 | | 10 | 3 | 6 | 5 | 16 | 5 |
|  |  | ASA | 16 | | 16 | | 16 | 8 | 8 | 12 | 26 | 8 |
|  |  | Pdisorder | 0 | | 0 | | 0 | 2 | 0 | 0 | 0 | 0 |
|  | Mutant | RSA | Buried | | Buried | | Buried | Buried | Buried | Buried | Exposed | Buried |
|  |  | RSA (%) | 12 | | 15 | | 25 | 2 | 6 | 6 | 32 | 5 |
|  |  | ASA | 19 | | 26 | | 39 | 4 | 8 | 10 | 85 | 13 |
|  |  | Pdisorder | 0 | | 0 | | 0 | 1 | 0 | 0 | 0 | 0 |
| mCSM | | | Destabilizing | | Destabilizing | | Destabilizing | Destabilizing | Destabilizing | Destabilizing | Destabilizing | Destabilizing |
| SDM | | | Destabilizing | | Destabilizing | | Destabilizing | Destabilizing | Destabilizing | Destabilizing | Destabilizing | Stabilizing |
| DUET | | | Destabilizing | | Destabilizing | | Destabilizing | Destabilizing | Destabilizing | Destabilizing | Destabilizing | Destabilizing |
| Change in size | | | Decrease | | Increase | | Increase | Decrease | Increase | Decrease | Increase | Increase |
| Change of charge | | | Unchanged | | Unchanged | | Unchanged | Unchanged | Neutral>Negative | Unchanged | Unchanged | Unchanged |
| Change in hydrophobicity | | | Unchanged | | Unchanged | | Increase | Unchanged | Decrease | Decrease | Decrease | Increase |
| Other impacts | | | The mutation will cause an empty space in the core of the protein. | | None | | The mutation will cause loss of hydrogen bonds in the core of the protein and as a result disturb correct folding. | The mutation converts the wild-type residue in a residue that does not prefer α-helices as secondary structure. | None | Mutation will cause an empty space in the core of the protein. | The residue being involved in a cysteine bridge, the mutation will have a severe effect on the 3D-structure. | Mutation will cause loss of hydrogen bonds in the core of the protein and as a result disturb correct folding. |
| **Amino Acid**  **Substitution** | | | **C328P** | | **G122R** | **I173N** | | **D91A** | **V258G** | **L212P** | **L254Q** | **A300V** |
| Mupro stability  prediction | | | Decrease | | Increase | Decrease | | Increase | Decrease | Decrease | Decrease | Decrease |
| Imutant2 | | | Decrease | | Decrease | Decrease | | Decrease | Decrease | Increase | Decrease | Increase |
| NetSurfP 3.0 prediction | Native | RSA | Exposed | | Buried | Exposed | | Buried | Buried | Exposed | Buried | Buried |
|  |  | RSA (%) | 49 | | 3 | 44 | | 2 | 5 | 55 | 14 | 1 |
|  |  | ASA | 81 | | 3 | 87 | | 3 | 7 | 104 | 28 | 1 |
|  |  | Pdisorder | 41 | | 0 | 0 | | 0 | 0 | 0 | 1 | 0 |
|  | Mutant | RSA | Exposed | | Buried | Exposed | | Buried | Buried | Exposed | Buried | Buried |
|  |  | RSA (%) | 62 | | 5 | 52 | | 1 | 7 | 45 | 18 | 1 |
|  |  | ASA | 99 | | 13 | 101 | | 2 | 7 | 72 | 40 | 2 |
|  |  | Pdisorder | 41 | | 0 | 0 | | 0 | 0 | 0 | 1 | 0 |
| mCSM | | | Destabilizing | | Destabilizing | Destabilizing | | Stabilizing | Destabilizing | Destabilizing | Destabilizing | Stabilizing |
| SDM | | | Destabilizing | | Destabilizing | Destabilizing | | Stabilizing | Destabilizing | Destabilizing | Destabilizing | Destabilizing |
| DUET | | | Destabilizing | | Destabilizing | Destabilizing | | Stabilizing | Destabilizing | Destabilizing | Destabilizing | Stabilizing |
| Change in size | | | Increase | | Increase | Increase | | Decrease | Decrease | Decrease | Increase | Increase |
| Change of charge | | | Unchanged | | Neutral>Positive | Unchanged | | Negative>Neutral | Negative>Neutral | Unchanged | Unchanged | Unchanged |
| Change in hydrophobicity | | | Unchanged | | Decrease | Decrease | | Increase | Unchanged | Unchanged | Decreased | Unchanged |
| Other impacts | | | None | | None | None | | Mutation will cause an empty space in the core of the protein. Mutation will cause loss of hydrogen bonds in the core of the protein and as a result disturb correct folding. | None | None | None | None |
