# Supplementary material for: In-silico characterization of deleterious non-synonymous SNPs in the human S1PR1 gene reveals structural instability and altered ligand affinity
Source: PLoS One. 2026 Feb 2;21(2):e0339370. doi: 10.1371/journal.pone.0339370 (PMC12863678; doi:10.1371/journal.pone.0339370)
Supplement: S4 Table — (DOCX) [file pone.0339370.s004.docx]

**S4 Table.** Evolutionary conservation prediction of S1PR1 protein through ConSurf.

| Residue and Position | Conservation Score | Prediction |
| --- | --- | --- |
| R120P | 9 | Highly conserved and exposed (f) |
| F125S | 9 | Highly conserved and buried (s) |
| C184Y | 9 | Highly conserved and buried (s) |
| Y198C | 8 | Very conserved and buried |
| L275P | 5 | Average conserved and buried |
