# Supplementary material for: In-silico characterization of deleterious non-synonymous SNPs in the human S1PR1 gene reveals structural instability and altered ligand affinity
Source: PLoS One. 2026 Feb 2;21(2):e0339370. doi: 10.1371/journal.pone.0339370 (PMC12863678; doi:10.1371/journal.pone.0339370)
Supplement: S5 Table — (DOCX) [file pone.0339370.s005.docx]

**S5 Table.** Protein model verification by PROCHECK and ERRAT.

| **Mutation** | **Quality Parameters** | | | | |
| --- | --- | --- | --- | --- | --- |
|  | PROCHECK Ramachandran plot | | | | ERRAT quality factor |
|  | Residues in most favoured regions (%) | Residues in additional allowed regions (%) | Residues in generously allowed regions (%) | Residues in disallowed regions (%) |  |
| Wild type | 94 | 6 | 0 | 0 | 95.4861 |
| R120P | 94 | 6 | 0 | 0 | 94.4251 |
| F125S | 94 | 6 | 0 | 0 | 95.4861 |
| C184Y | 95.1 | 4.6 | 0.4 | 0 | 95.0877 |
