# Supplementary material for: In-silico characterization of deleterious non-synonymous SNPs in the human S1PR1 gene reveals structural instability and altered ligand affinity
Source: PLoS One. 2026 Feb 2;21(2):e0339370. doi: 10.1371/journal.pone.0339370 (PMC12863678; doi:10.1371/journal.pone.0339370)
Supplement: S6 Table — (DOCX) [file pone.0339370.s006.docx]

**S6 Table.** Forty-six key residues predicted in the S1PR1 protein.

| **Key residues (N=46)** | **Residues selected for molecular docking** |
| --- | --- |
| Y29, K34, L35, N36, E42, I45, T48, S49, F52, Y98, N101, L102, S105, G106, A107, T109, Y110, W117, R120, E121, M124, F125, L128, S129, V132, F133, L174, V194, L195, P196, I203, C206, T207, V209, F210, L213, W269, L272, F273, L275, L276, L290, F291, A293, E294, and L297 | R120P and F125S |
