# Supplementary material for: In-silico characterization of deleterious non-synonymous SNPs in the human S1PR1 gene reveals structural instability and altered ligand affinity
Source: PLoS One. 2026 Feb 2;21(2):e0339370. doi: 10.1371/journal.pone.0339370 (PMC12863678; doi:10.1371/journal.pone.0339370)
Supplement: S1 Fig — SNP datasets were retrieved from dbSNP-NCBI, followed by functional impact assessment using multiple prediction tools (SIFT, PolyPhen-2, PROVEAN, etc.). Protein stability was analyzed with MUpro, I-Mutant 2.0, and NetSurfP-3.0, while domain identification was performed using Pfam. Evolutionary and conservation analyses were conducted with MEGA11, Iroki, and ConSurf. Homology modeling and model validation were carried out using SWISS-MODEL, PROCHECK, and ERRAT. Finally, molecular docking and molecular dynamics simulations were performed using PyRx and Schrödinger. (DOCX) [file pone.0339370.s007.docx]

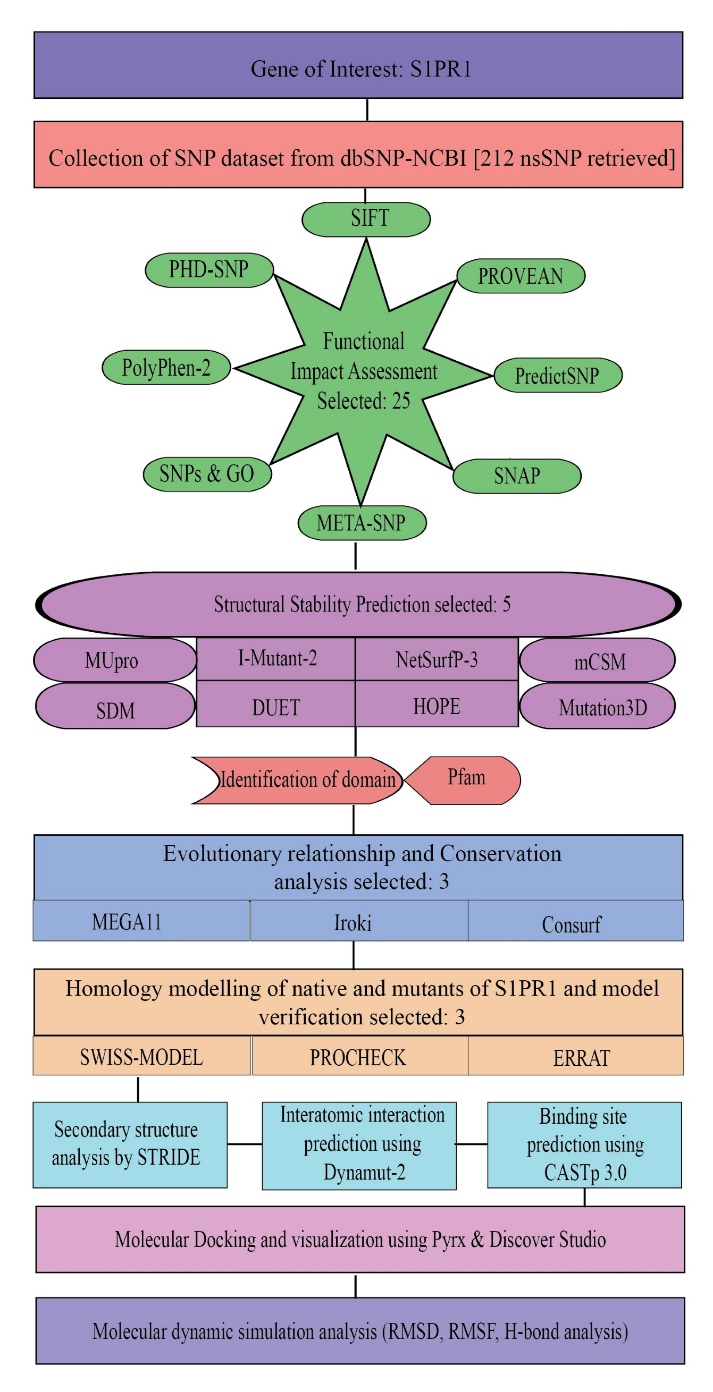


**S1 Fig. Workflow for evaluating the functional and structural consequences of S1PR1 gene variants.** SNP datasets were retrieved from dbSNP-NCBI, followed by functional impact assessment using multiple prediction tools (SIFT, PolyPhen-2, PROVEAN, etc.). Protein stability was analyzed with MUpro, I-Mutant 2.0, and NetSurfP-3.0, while domain identification was performed using Pfam. Evolutionary and conservation analyses were conducted with MEGA11, Iroki, and ConSurf. Homology modeling and model validation were carried out using SWISS-MODEL, PROCHECK, and ERRAT. Finally, molecular docking and molecular dynamics simulations were performed using PyRx and Schrödinger.
