# Supplementary material for: In-silico characterization of deleterious non-synonymous SNPs in the human S1PR1 gene reveals structural instability and altered ligand affinity
Source: PLoS One. 2026 Feb 2;21(2):e0339370. doi: 10.1371/journal.pone.0339370 (PMC12863678; doi:10.1371/journal.pone.0339370)
Supplement: S2 Fig — Analysis highlights three SNPs namely R120P, F125S, and C184Y as highly conserved positions predicted to exert the greatest impact on the structural integrity of the protein. These residues are marked within rectangular boxes for emphasis. (DOCX) [file pone.0339370.s008.docx]

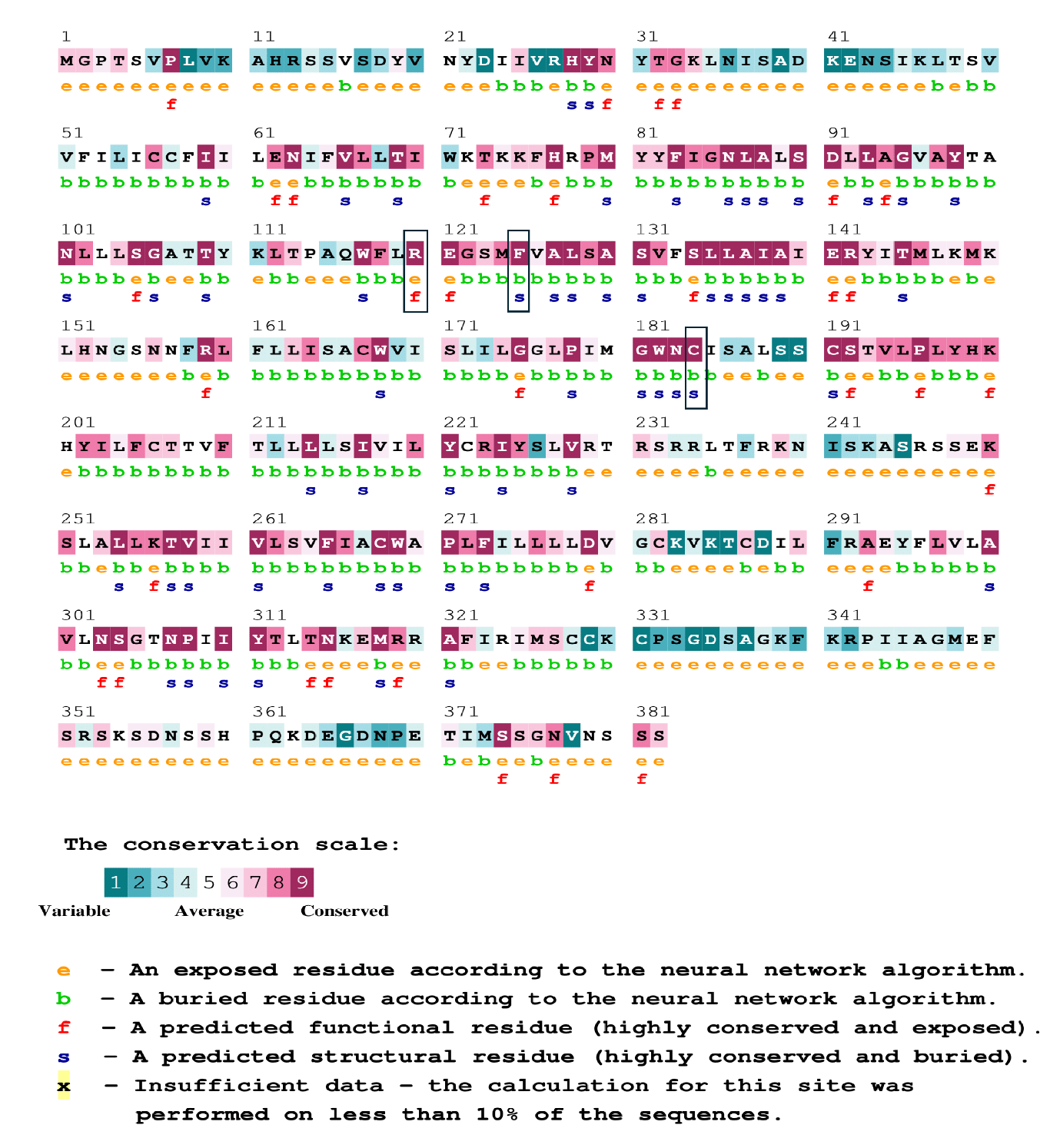


**S2 Fig.** Conservation profile of the human S1PR1 protein. Analysis highlights three SNPs namely R120P, F125S, and C184Y as highly conserved positions predicted to exert the greatest impact on the structural integrity of the protein. These residues are marked within rectangular boxes for emphasis.
