# Supplementary material for: In-silico characterization of deleterious non-synonymous SNPs in the human S1PR1 gene reveals structural instability and altered ligand affinity
Source: PLoS One. 2026 Feb 2;21(2):e0339370. doi: 10.1371/journal.pone.0339370 (PMC12863678; doi:10.1371/journal.pone.0339370)
Supplement: S3 Fig — Human S1PR1 gene shares a close evolutionary relationship with its homologs in Pan troglodytes and Pongo abelii. (DOCX) [file pone.0339370.s009.docx]

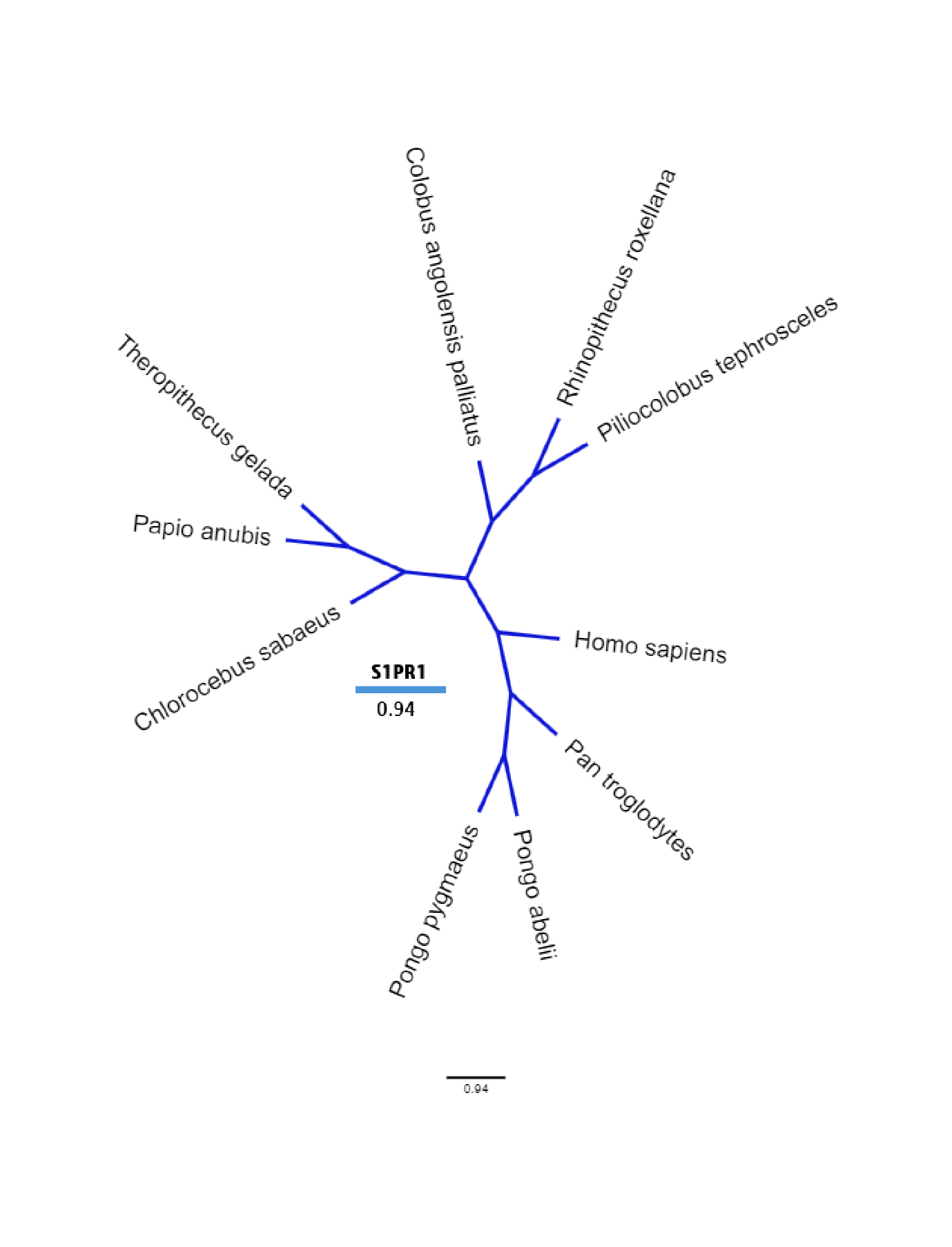


**S3 Fig.** Evolutionary phylogenetic analysis of the human *S1PR1* gene. Human *S1PR1* gene shares a close evolutionary relationship with its homologs in *Pan troglodytes* and *Pongo abelii*.
