# Supplementary material for: In-silico characterization of deleterious non-synonymous SNPs in the human S1PR1 gene reveals structural instability and altered ligand affinity
Source: PLoS One. 2026 Feb 2;21(2):e0339370. doi: 10.1371/journal.pone.0339370 (PMC12863678; doi:10.1371/journal.pone.0339370)
Supplement: S4 Fig — (DOCX) [file pone.0339370.s010.docx]

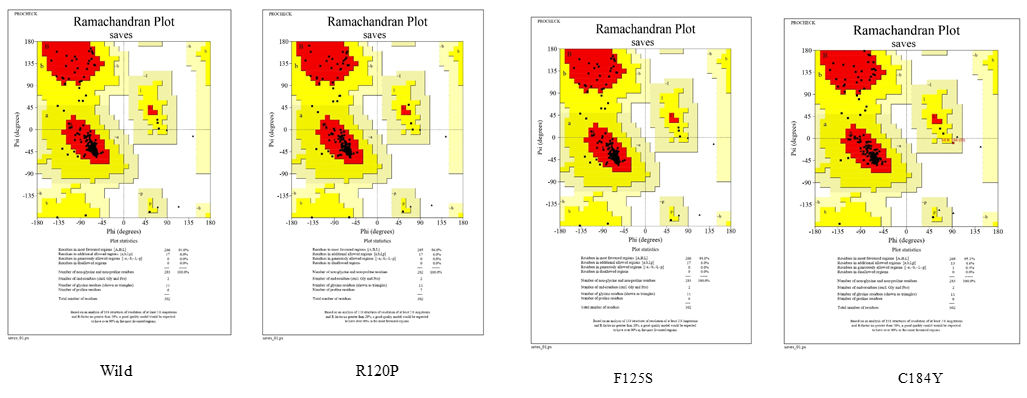


**S4 Fig.** Ramachandran plots showing the backbone dihedral angle distributions of wild-type and mutant proteins, illustrating the conformational quality, stereochemical validity, and structural stability of the modeled protein structures.
