# Supplementary material for: In-silico characterization of deleterious non-synonymous SNPs in the human S1PR1 gene reveals structural instability and altered ligand affinity
Source: PLoS One. 2026 Feb 2;21(2):e0339370. doi: 10.1371/journal.pone.0339370 (PMC12863678; doi:10.1371/journal.pone.0339370)
Supplement: S5 Fig — (E) Indicates the used legends of secondary structure icons. Changes in structural elements are shown in rectangular boxes. (DOCX) [file pone.0339370.s011.docx]

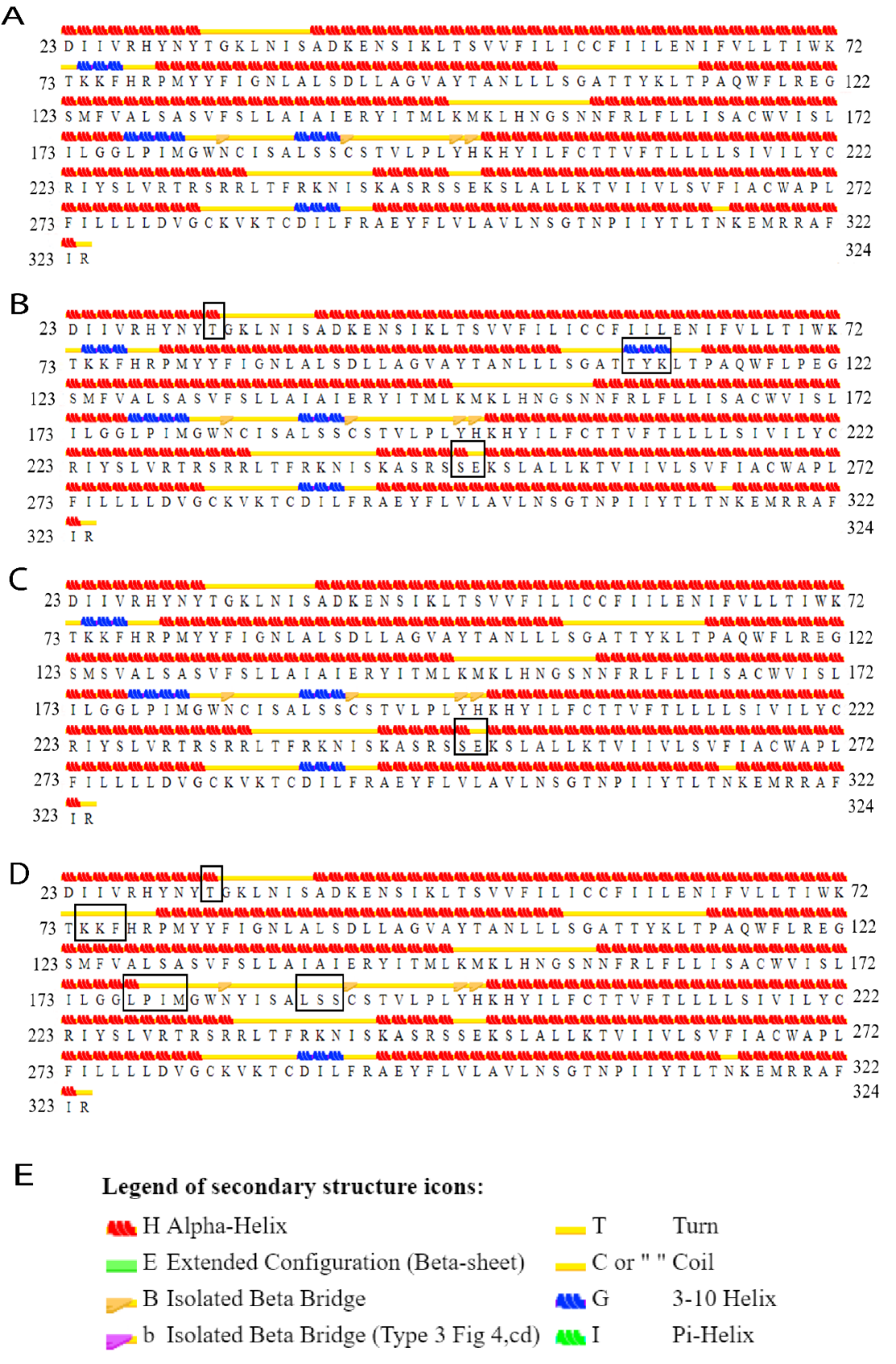


**S5 Fig.** Secondary structure analysis of wild type (A) and three selected mutants (B) R120P, (C) F125S and (D) C184Y by STRIDE program. (E) Indicates the used legends of secondary structure icons. Changes in structural elements are shown in rectangular boxes.
